# Quantitative Analysis and Risk Assessment of Polycyclic Aromatic Hydrocarbons Using Gas Chromatography–Mass Spectrometry from Herbs and Spices Distributed in South Korea

**DOI:** 10.3390/foods14213595

**Published:** 2025-10-22

**Authors:** Seung-Hyun Sa, Kyung-Jik Lim, Han-Seung Shin

**Affiliations:** Department of Food Science and Biotechnology, Dongguk University-Seoul, 32, Dongguk-ro, Ilsandong-gu, Goyang-si 10326, Gyeonggi-do, Republic of Korea; sasa0322@naver.com (S.-H.S.); kyung9209@naver.com (K.-J.L.)

**Keywords:** polycyclic aromatic hydrocarbon, herb, spice, analytical validation, risk assessment

## Abstract

In this study, four polycyclic aromatic hydrocarbons (4PAHs: benz[a]anthracene, chrysene, benzo[b]fluoranthene, and benzo[a]pyrene (BaP)) were quantified in 110 herb and spice products marketed in South Korea. A validated gas chromatography–mass spectrometry (GC-MS) method achieved high sensitivity with limits of detection (LOD) ranging from 0.08 to 0.18 µg/kg and limits of quantification (LOQ) ranging from 0.24 to 0.55 µg/kg, with recoveries consistent with the Association of Official Analytical Chemists guidelines. Among the tested items, oregano contained the highest BaP concentrations with 7.13 µg/kg, while overall concentrations of the sum of 4PAHs remained below European Union limits. The BaP-based toxic equivalent (TEQ_BaP_) and the toxic equivalent for the sum of 4PAHs (TEQ_Σ4PAHs_) were 7.13 and 7.50 µg/kg, respectively, with oregano showing the highest values. Risk assessment using the margin of exposure (MOE) showed all values exceeded 10^6^, indicating negligible health concern. These findings provide a basis for continuous monitoring and regulatory management of PAHs in herbs and spices.

## 1. Introduction

Polycyclic aromatic hydrocarbons (PAHs) are non-polar organic compounds consisting of two or more fused benzene rings, generated during the incomplete combustion of organic matter [1]. PAHs originate from both natural sources, such as wildfires, and anthropogenic activities, including coal combustion, automobile exhaust, and tobacco smoke [2,3]. More than 90% of human exposure to PAHs arises from dietary intake, reaching up to 98% in non-smokers [4]. Due to their non-polar characteristics and resistance to degradation, environmental PAHs tend to bioaccumulate within the organic matrices of aquatic organisms, animal products, and plant-based raw materials [5]. In addition, industrial processing methods such as heating and drying, as well as household cooking practices such as grilling and roasting, promote the formation of new PAHs and contribute to further contamination of foods [6,7]. Consequently, dietary exposure to PAHs represents the combined effect of environmental accumulation and contamination occurring during processing and cooking.

After absorption into the body, PAHs undergo metabolic activation to form chemically reactive intermediates, which can subsequently establish covalent bonds with critical intracellular macromolecules such as DNA, leading to genetic damage and thereby promoting mutations and tumor development [8]. The International Agency for Research on Cancer (IARC) has classified benzo[a]pyrene (BaP) as a Group 1 carcinogen (carcinogenic to humans), whereas benz[a]anthracene (BaA), chrysene (CHR), and benzo[b]fluoranthene (BbF) are designated as Group 2B (possibly carcinogenic to humans) [9]. Based on these carcinogenic properties, the European Food Safety Authority (EFSA) concluded that BaP alone is not a suitable indicator for the occurrence and toxicity of PAHs in food. Consequently, EFSA recommends using the sum of these 4PAHs (Σ4PAHs) as a more appropriate marker. This 4PAH-based approach is now widely employed in risk assessments, such as the margin of exposure (MOE), underscoring the need for targeted evaluations in high-risk food groups [10].

Herbs and spices represent a major source of PAH contamination in foods due to both the intrinsic characteristics of plant-based raw materials and their processing methods. Residual PAHs in the environment can accumulate within plant tissues through root uptake, stomatal diffusion, and particle deposition [2,11]. Moreover, high-temperature processes, such as drying, may lead to the formation of new PAHs or to additional contamination, either through contact with combustion by-products or exposure to elevated thermal conditions [12,13].

Monitoring studies conducted outside of South Korea have consistently shown that PAHs are frequently detected in herb and spice samples. A survey conducted by the UK Food Standards Agency (FSA) reported mean concentrations of 3 µg/kg for BaP and 17.76 µg/kg for Σ4PAHs across 25 herb and spice samples [13]. In Belgium, BaP was detected in 56% of commercially available spices, with Σ4PAHs concentrations reaching up to 164 µg/kg [14]. In China, Σ4PAHs levels in spices ranged from non-detectable to as high as 252.97 µg/kg [15]. However, data on the situation in South Korea are highly limited. A literature review using relevant keyword combinations in PubMed found no prior studies on PAH contamination in herbs and spices distributed in South Korea. This absence of research indicated the lack of scientific evidence to assess potential exposure levels among Korean consumers and to establish management standards. Therefore, this study fills an important research gap and possesses significant scientific necessity by providing fundamental data essential for future policy development in this area.

In this study, we quantitatively evaluated the 4PAHs contamination in 110 herb and spice products distributed in South Korea, and the results were subsequently compared with international guideline values to provide a scientific basis for future regulation and management. A gas chromatography–mass spectrometry (GC-MS)-based analytical method was validated, and the incidence of detection, as well as concentration distributions, were determined for each product. By integrating domestic consumption scenarios, we further estimated toxic equivalent quantity (TEQ) and MOE to conduct a quantitative risk assessment. This study is significant in that it presents a comprehensive approach combining quantitative monitoring with risk assessment for herbs and spices. This approach offers empirical evidence and prioritization insights to support food safety management and regulatory decision-making.

## 2. Materials and Methods

### 2.1. Chemicals and Materials

Standards for the analysis of 4PAHs were obtained from Sigma-Aldrich (St. Louis, MO, USA). They included BaA (CAS No. 56-55-3), CHR (CAS No. 218-01-9), BbF (CAS No. 205-99-2), and BaP (CAS No. 50-32-8). To increase the precision of the analysis, internal standards (IS) CHR-*d_12_* (CAS No. 1719-03-5) and BaP-*d_12_* (CAS No. 63466-71-7) were also obtained from Sigma-Aldrich. All standards and IS were classified for analysis.

Among the solvents and other chemicals, dichloromethane (DCM, CAS No. 75-09-2) and n-hexane (CAS No. 110-54-3) were purchased from Honeywell International Inc. (Charlotte, NC, USA). Ethanol (CAS No. 64-17-5) and 95% potassium hydroxide (KOH, CAS No. 1310-58-3) were obtained from Samchun Pure Chemical Co., Ltd. (Pyeongtaek, Republic of Korea). Water was obtained from a Milli-Q Water Purification System (Billerica, MA, USA). Sodium sulfate (Na_2_SO_4_, CAS No. 7757-82-6), used as the desiccant, was purchased from Daejung Chemicals & Metals Co., Ltd. (Siheung, Republic of Korea). For sample purification, solid-phase extraction (SPE) was performed using Sep-Pak Florisil cartridges from Waters Corp (Milford, MA, USA). For the final filtration, a 0.45 μm polytetrafluoroethylene (PTFE) membrane filter supplied by Agilent Technologies, Inc. (Santa Clara, CA, USA) was used.

### 2.2. Sample Preparation for 4PAHs Evaluation

**Figure 1:**
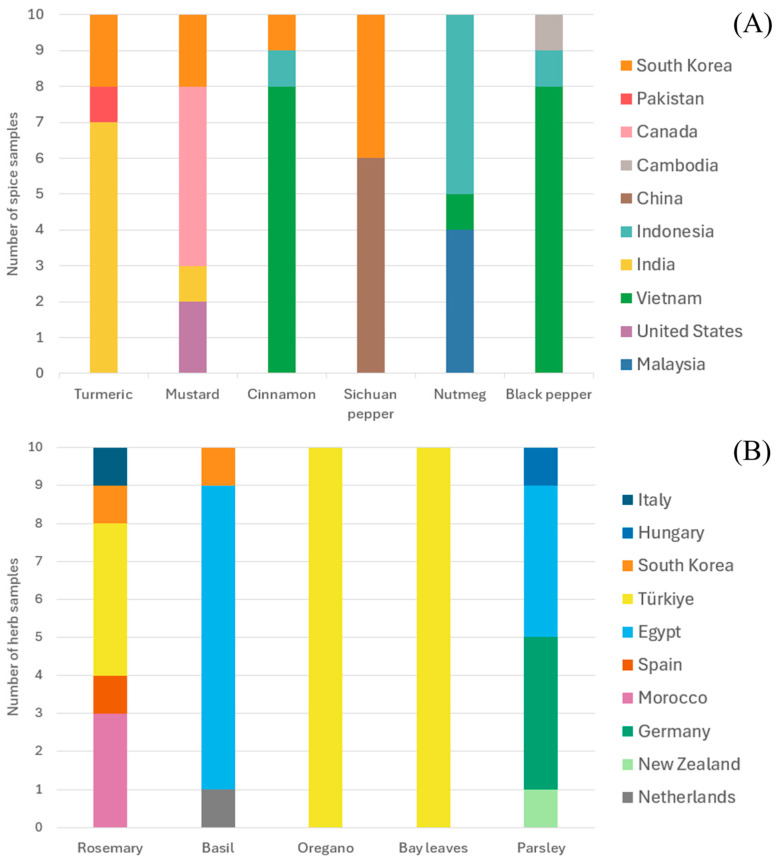
Country of origin for the (**A**) spice products and (**B**) herb products distributed in South Korea.

Herb and spice samples were procured from hypermarkets, small discount stores, and online retailers. Spices included mustard, nutmeg, black pepper, Sichuan pepper, cinnamon, and turmeric. Herbs included basil, oregano, parsley, rosemary, and bay leaves. Commodity selection was based on domestic market prevalence, as reflected in UN Comtrade/WITS import volumes, and added items frequently included in previous international PAH research. This ensured representativeness for both Korean consumption patterns and cross-study comparability. The origins of herb and spice products distributed in the South Korean markets are shown in Figure 1. Origin was taken as the country declared on the product label. The analyzed spices originated from 10 countries, including South Korea, Pakistan, Canada, Cambodia, China, Indonesia, India, Vietnam, the United States, and Malaysia. The herb products originated from various countries such as Italy, Hungary, South Korea, Turkey, Egypt, Spain, Morocco, Germany, New Zealand, and the Netherlands. Samples were processed using a versatile blender (model SBL365AB, Ningbo Kajafa Electric Appliance Co., Ltd., Ningbo, China) equipped with stainless steel blades. This grinding process resulted in a fine powder with an average particle size of approximately 250 μm. A total of 10 g of ground herb and spice products was used for subsequent analysis.

### 2.3. Extraction and Clean-Up for Preparation

The analysis of 4PAHs in this study followed the BaP General Test Method established by the Ministry of Food and Drug Safety (MFDS) in South Korea [16]. After weighing 10 g of the homogenized sample accurately in a 300 mL flask, 1 mL of an IS solution containing CHR-*d_12_* and BaP-*d_12_* was spiked, such that the final concentration in the sample was 100 µg/kg.

For alkaline hydrolysis and efficient 4PAHs extraction, 100 mL of 1 M KOH was added and refluxed at 80 °C for 3 h. After cooling the extract to room temperature, 50 mL of n-hexane was added via a reflux cooling tube. The resulting alkaline hydrolysate was washed with 50 mL of a mixture of ethanol/n-hexane (1:1, *v*/*v*), and all the solutions were combined into the separatory funnel. Hexane layers from two successive extractions, each with 50 mL of n-hexane, were combined. The n-hexane extract was washed three times with 50 mL aliquots of deionized water to remove residual polar impurities.

The purified n-hexane layer was dried over Na_2_SO_4_, transferred to a 250 mL flask, and concentrated by rotary evaporation at 40 °C. For further purification, SPE was performed. To this end, each Sep-Pak Florisil cartridge (6 cc Vac Cartridge, 50−200 µm) was conditioned with 10 mL of DCM and 20 mL of n-hexane. The concentrated extract was loaded onto the conditioned cartridge. The 4PAHs were eluted with 10 mL of n-hexane, followed by 20 mL of n-hexane/DCM (3:1, *v*/*v*). The eluate was evaporated to dryness under nitrogen gas at 40 °C and reconstituted in 1 mL DCM. The solution was filtered through a 0.45 μm PTFE membrane filter and transferred to a 2 mL amber vial. Sample volumes of 1 µL were injected into the GC-MS system for 4PAHs quantification.

### 2.4. Quantitative Analysis of 4PAHs by GC-MS

The extracted samples were analyzed using an Agilent 7820A gas chromatograph coupled to a 5975 mass-selective detector (MSD; Agilent Technologies, Inc.). A 1 μL aliquot of each extract was injected in splitless mode at an inlet temperature of 310 °C, allowing the analytes to enter the column without thermal decomposition. Separation was achieved on a Zebron ZB-PAH-SeleCT capillary column (30 m × 0.25 mm, 0.20 μm film thickness, Torrance, CA, USA). Helium (99.99% purity) was used as the carrier gas, and the flow rate of the carrier gas was kept constant at 1.2 mL/min to ensure reproducible retention times. The column oven temperature was held at 80 °C for the initial minute to optimize the separation of initially eluted compounds. The temperature was then gradually increased to 245 °C at a rate of 6 °C/min, followed by a further increase to 270 °C at 30 °C/min and held for 13 min to ensure complete elution of the high-molecular-weight 4PAHs. A post-run temperature of 310 °C was held for 10 min to remove residual contaminants and re-equilibrate the column. The mass spectrometer maximized detection sensitivity and selectivity for target PAH compounds by simultaneously using an electron ionization (EI) mode and a selective ion monitoring (SIM) scheme. The ion source and quadrupole temperatures were maintained at 250 and 150 °C, respectively, to enhance ion stability and the precision of the quantitative analysis. Peak integration and area calculation were performed in the MSD Chem Station Data Analysis Application (Agilent Technologies, Inc.). For each analyte and internal standard, areas were obtained from the extracted-ion chromatograms and used to construct calibration curves based on analyte-to-IS area ratios for quantification.

### 2.5. Validation of Analytical Method

Validation for 4PAHs quantification was performed in accordance with the Association of Official Analytical Chemists, assessing accuracy, precision, linearity, limit of detection (LOD), and limit of quantification (LOQ). Calibration curves were constructed at five concentrations (1, 2, 5, 10, and 20 µg/kg). LOD was calculated based on a signal-to-noise ratio (S/N) of 3.3. The LOQ was calculated based on an S/N of 10. Mustard powder and parsley were selected as representative samples of spices and herbs, respectively, due to their frequent consumption. In addition, these commercially available samples, in which 4PAHs were not detected, minimized potential matrix effects during validation. Recovery efficiency was evaluated by spiking the representative samples with 4PAHs standards (at five concentration levels) and two IS (100 µg/kg). The recovery rate was calculated by dividing the difference in 4PAHs content between spiked and non-spiked samples by the spiked level. Accuracy and precision were evaluated by intra- and inter-day replicate analyses of spiked samples. Intra-day precision was assessed by analyzing the same sample three times within a single day, while inter-day precision was evaluated across different days to confirm the robustness and reproducibility of the analytical method.

### 2.6. Evaluation of TEQ in Herb and Spice

The toxicity of PAHs varies across compounds, making relative toxicity the appropriate metric for comparison. In this study, TEQ was used to characterize potential risks from the consumption of herbs and spices. The toxic equivalency factor (TEF) is an index that defines the carcinogenicity of each PAH relative to BaP (TEF = 1) [17]. For each sample, TEQ was calculated as follows:

$$TEQ=\sum_{i=1}^{n}\left[C_{i}\right]\times {TEF}_{i}$$

where *C_i_* is the concentration of PAHs (µg/kg), and TEF*_i_* is the toxic equivalency factor for each PAH.

### 2.7. Exposure and Risk Assessment of PAHs in Herbs and Spices

Exposure to PAHs from herbs and spices was evaluated using the estimated daily intake (EDI, ng/kg_bw_/day). EDI was calculated as follows:

$$EDI(ng/{kg}_{bw}/day)=C\times [IR/BW]$$

where *C* is the TEQ concentration of BaP and 4PAHs in the food, the ingestion rate (*IR*) is the average daily intake (g/day) from the 2023 Korea National Health and Nutrition Examination Survey (KNHANES) database, and *BW* is the mean body weight (66.83 kg) from the 2023 Health Examination Statistical Yearbook [18,19].

For risk characterization, the EFSA approach to MOE was used as follows:

$$MOE={BMDL}_{10}/EDI$$

where BMDL_10_ is the 95% lower confidence limit of the benchmark dose corresponding to a 10% response. We used BMDL_10_ values of 0.07 mg/kg_bw_/day for BaP and 0.34 mg/kg_bw_/day for 4PAHs, respectively [20].

### 2.8. Statistical Analysis

Each experiment was performed in three repetitions, and the results were presented as the mean ± standard deviation (SD). Concentrations below the LOQ were replaced with zero. The distribution was described using the median accompanied by the interquartile range (IQR). Data processing was carried out using Microsoft Office Excel 2025 (Microsoft Corp., Redmond, WA, USA).

## 3. Results and Discussion

### 3.1. GC-MS Method Validation for Quantification of 4PAHs in Herbs and Spices

**Figure 2:**
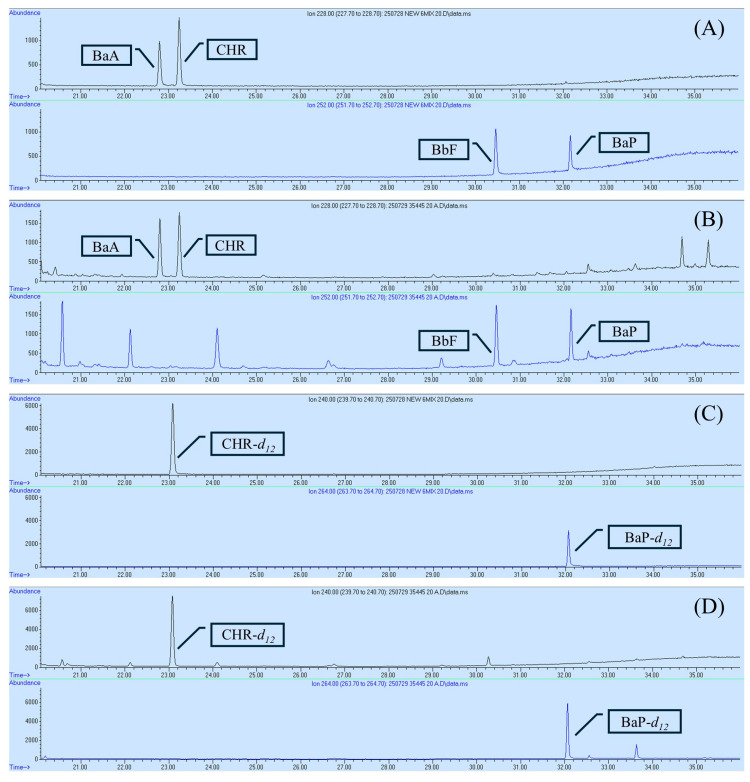
GC-MS chromatograms of BaA, CHR, BbF, BaP, and IS standards: (**A**) blank sample with 4PAH standards, (**B**) spiked sample containing 4PAH standards, (**C**) IS in a blank sample, and (**D**) spiked sample containing IS.

GC-MS chromatograms for the standards of BaA, CHR, BbF, BaP, and IS in blank and spiked samples are provided in Figure 2. For the validation of 4PAHs analysis, key parameters including linearity, accuracy, precision, LOD, and LOQ were evaluated in accordance with the Association of Official Analytical Chemists (AOAC) [21]. All target PAHs were adequately separated within 35 min, and calibration curves were constructed based on the ratio of each PAH peak area to that of the IS (Table 1). All analytes demonstrated linearity, with correlation coefficients (R^2^) exceeding 0.998. The LOD and LOQ ranged from 0.08 to 0.18 and 0.24 to 0.55 µg/kg, respectively, in spices, and from 0.11 to 0.17 and 0.32 to 0.51 µg/kg, respectively, in herbs. These values fall within the requirements set by European Commission Regulation No. 836/2011, which stipulates that the LOD must be below 0.30 µg/kg and the LOQ below 0.90 µg/kg [22]. Previous investigations of herbs and spices, LOD were generally comparable to those in the present study, but BbF exhibited a comparatively high LOD of 2.5 μg/kg. The LOQ for CHR (1.21 μg/kg) and BaP (1.13 μg/kg) were also higher than in our work. Notably, the LOQ for BbF reached 8.41 μg/kg, whereas our method delivered uniformly low LODs and LOQs across all 4PAHs, demonstrating stable analytical performance [23]. In another study, the reported LOD was 0.7 μg/kg, while the present method achieved LODs ≤ 0.2 μg/kg for all targets, demonstrating superior sensitivity [24]. Therefore, the validated GC-MS method provides sufficient sensitivity for the quantification of the 4PAHs.

Table 2 presents the recovery rates evaluated to validate the method for the 4PAHs. In spices, the lowest recovery was 88.6% for BaA at 1 µg/kg, while the highest was 116.5% for BaA at 20 µg/kg. In herbs, the lowest recovery was 85.0% for BbF at 5 µg/kg, and the highest was 112.9% for BaA at 20 µg/kg. A previous report showed recoveries in the range of 72−107%, with CHR exhibiting the lowest value of 72% at 1 µg/kg [25]. In contrast, our study showed no decrease in recovery at low concentrations, and recoveries consistently satisfied the recommended range (70−120%). In another study, the means of recoveries were 80% for BaA, 84% for CHR, 80% for BbF, and 110% for BaP [23]. While the present study also shows some variability, the recoveries are closer to 100% across the analytes than those reported previously. The recoveries in the 110% range observed at certain higher spiking levels can be attributed to matrix-induced response enhancement, commonly reported for essential-oil-rich matrices [26]. Taken together, these results corroborate the validity of this analytical method for quantifying the 4PAHs.

The accuracy and precision of the method were evaluated through intra- and inter-day repeatability tests (Table 3). In the spice matrix represented by mustard, intra-day results for the 4PAHs showed accuracies ranging from 88.6% to 116.6% with RSDs of 0.3∓1.7%, while inter-day results ranged from 88.1% to 116.6% with RSDs of 0.1∓2.0%. In the herb matrix represented by parsley, intra-day accuracies ranged from 85.0% to 109.6% with RSDs of 0.0∓1.8%, and inter-day accuracies ranged from 85.0% to 112.6% with RSDs of 0.0∓1.2%. Compared with the 2∓15% RSDs reported in a previous study, the present study maintained RSDs ≤ 2% under all tested conditions, resulting in a markedly narrower variability [25]. This indicates that the method provides sufficient repeatability and consistency even in complex herb and spice matrices. All values were within the recommended ranges, confirming that the analytical method provides consistent and reliable performance for the quantification of 4PAHs.

### 3.2. Evaluation of 4PAHs Content in Herbs and Spices

This study analyzed six types of spices and five types of herbs. The spices analyzed were mustard, nutmeg, black pepper, Sichuan pepper, cinnamon, and turmeric, while the herbs were basil, oregano, parsley, rosemary, and bay leaves. For each type, 10 different commercial samples were collected, resulting in a total of 110 samples for monitoring. For each sample, the concentrations of individual PAHs and the total concentration of the 4PAHs (Σ4PAHs) are summarized in Table 4. Among the herbs, bay leaves showed the highest levels of BaA and CHR at 5.12 ± 5.33 and 7.61 ± 6.50 µg/kg, respectively, whereas rosemary exhibited the lowest levels at 0.07 ± 0.22 and 0.36 ± 0.69 µg/kg, respectively. The low PAH concentrations observed in rosemary are consistent with a previous study, which reported that rosemary contained the lowest PAH levels among 11 tested herbs and spices [27]. Among the spices, cinnamon had the highest concentrations of BaA and CHR at 3.39 ± 4.86 and 6.98 ± 5.27 µg/kg, respectively. Mustard exhibited the lowest levels of BaA at 0.18 ± 0.45 µg/kg, and turmeric exhibited the lowest levels of CHR at 0.20 ± 0.42 µg/kg, respectively.

For BaP, cinnamon exhibited the highest concentration at 6.18 ± 3.46 µg/kg among the spices, while oregano had the highest concentration at 7.13 ± 6.68 µg/kg among the herbs. This finding is consistent with previous studies, which reported that oregano contained high levels of BaP compared with various other herbs and spices [28]. BaP levels in samples other than cinnamon and oregano were approximately 1.00 µg/kg. Meanwhile, BbF was not detected in nutmeg, turmeric, and parsley, suggesting compound-specific detection variability.

The mean concentrations of BaA, CHR, BbF, and BaP in spices were 1.37, 2.44, 0.87, and 1.50 µg/kg, respectively, and their corresponding values in herbs were 1.34, 2.37, 1.20, and 2.18 µg/kg, respectively, indicating no substantial differences between the two groups. For the Σ4PAHs, cinnamon and Sichuan pepper had the highest values of 4.83 and 1.80 µg/kg, respectively, among the spices, whereas bay leaves and oregano exhibited the highest concentrations of 3.60 and 2.94 µg/kg, respectively, among the herbs. Previous studies have reported higher levels than those observed in the present work, with Σ4PAHs concentrations of 13.86 µg/kg in cinnamon and 3.74 µg/kg in oregano [13,25]. Oregano and cinnamon are reported to be spices rich in sesquiterpenes [29,30,31,32]. These components are a major group of compounds that determine the aroma and flavor in many spices [33]. In particular, β-caryophyllene, which is commonly found in both products, is known to be a sesquiterpene that is more sensitive to pyrolysis, and it has been reported to lead to the formation of PAHs under high-temperature conditions [34]. This mechanism suggests that the high levels of PAH contamination in oregano and cinnamon may be the result of a combination of their processing methods and inherent chemical compositions.

**Figure 3:**
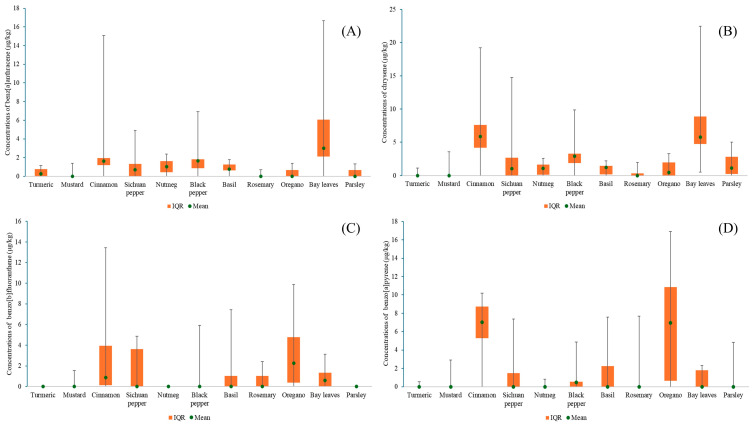
Distribution of PAH concentrations in herb and spice products (median, IQR, range): (**A**) concentrations of benz[a]anthracene, (**B**) concentrations of chrysene, (**C**) concentrations of benzo[b]fluoranthene, and (**D**) concentrations of benzo[a]pyrene.

However, considerable variability was observed across individual items, with SDs comparable to or even exceeding the means, as in the case of BaP in rosemary (0.77 ± 2.43 µg/kg) and mustard (0.29 ± 0.92 µg/kg). The large SDs reflect substantial variability in the data, with values ranging from not detected (ND) to elevated concentrations. Accordingly, the mean alone may not adequately capture the central tendency of the distribution. Therefore, Table 5 presents the medians and detection rates, which better describe the distribution characteristics and highlight commodities with consistently high contamination levels. Figure 3 complements this by presenting box plots of PAH concentrations (median, interquartile range), offering a visual summary of dispersion and enabling rapid comparison. Based on the medians, BaP concentrations were high in cinnamon (7.02 µg/kg, IQR 5.26−8.74 µg/kg) among spices, and in oregano (6.95 µg/kg, IQR 0.64−10.84 µg/kg) among herbs. Detection rates were consistently high for CHR, reaching 100% in bay leaves, 90% in black pepper, and 90% in cinnamon, which is consistent with the literature. Previous monitoring studies of various foods have also reported CHR as one of the most frequently detected PAHs [35,36], and it has often been observed even in samples where BaP was absent [20].

The European Union (EU) has established the primary international benchmark for PAHs in herbs and spices, setting maximum limits at 10 µg/kg for BaP and 50 µg/kg for Σ4PAHs [37]. These legally binding standards apply across all EU member states and are also adopted by the United Kingdom and Türkiye [38,39]. However, most countries have not established product-specific limits for PAHs in herbs and spices. For example, Australia and the United States do not set maximum levels for PAHs in herbs and spices. Also, China sets BaP limits for some foods, such as edible oil and aquatic products, but does not specify limits for herbs and spices [40]. In Korea, PAH limits are specified for herbal medicinal ingredients, but not for culinary herbs and spices [41]. These regulatory gaps highlight the critical need for base data to guide risk management and the establishment of standards in non-EU markets.

The mean concentrations for most commodities in the present study were below these regulatory limits; a potential risk of non-compliance was identified for certain high-risk items. For instance, the upper quartile (Q3) of BaP in oregano reached 10.84 µg/kg, slightly exceeding the EU limit. This pattern of high contamination is consistent with previous surveys in the United Kingdom (up to 14.49 µg/kg) and China (average of 23.30 µg/kg), confirming that certain herbs are a significant international concern [13,42].

### 3.3. Interpretation of the Origins of PAH Contamination in Herbs and Spices

The occurrence of PAHs in herbs and spices is influenced by a combination of environmental factors, plant matrix characteristics, and drying processes [43,44]. The specific origins of environmental PAHs are closely connected with the geographical and environmental context of the cultivation area. For instance, low-molecular-weight PAHs present in the soil due to historical pollution or atmospheric deposition can be taken up through the plant root system [45]. Conversely, high-molecular-weight, particle-bound PAHs are abundant in the atmosphere, and their primary contamination pathway is direct atmospheric deposition onto leaf surfaces [46]. In particular, the hydrophobicity of leaf surfaces enhances PAH uptake. Previous studies have shown that PAH accumulation increases with the development of leaf surface structures and with greater overall surface area [47,48]. The extent of deposition is influenced by the proximity of the cultivation areas to anthropogenic emission sources such as industrial regions, high-traffic roads, and urban centers [49,50]. Furthermore, local and seasonal factors, like the burning of agricultural waste or the use of specific heating fuels, can increase atmospheric PAH levels [51,52,53]. Finally, climatic and meteorological conditions govern the partitioning and deposition efficiency of gas-phase PAHs, which can lead to different contamination levels even in regions with similar emission sources [54,55].

Against this backdrop, the subsequent processing stage becomes a critical cause of PAHs accumulation. Drying is a crucial step in herb and spice processing, as it reduces moisture content and preserves quality [56]. However, prolonged drying by the sun and air can promote PAH accumulation from the surrounding environment. Also, fuel-based drying may lead to additional deposition through smoke contact and surface adsorption [57]. Thus, process-specific factors and environmental conditions likely determine the final PAHs in herbs and spices.

**Figure 4:**
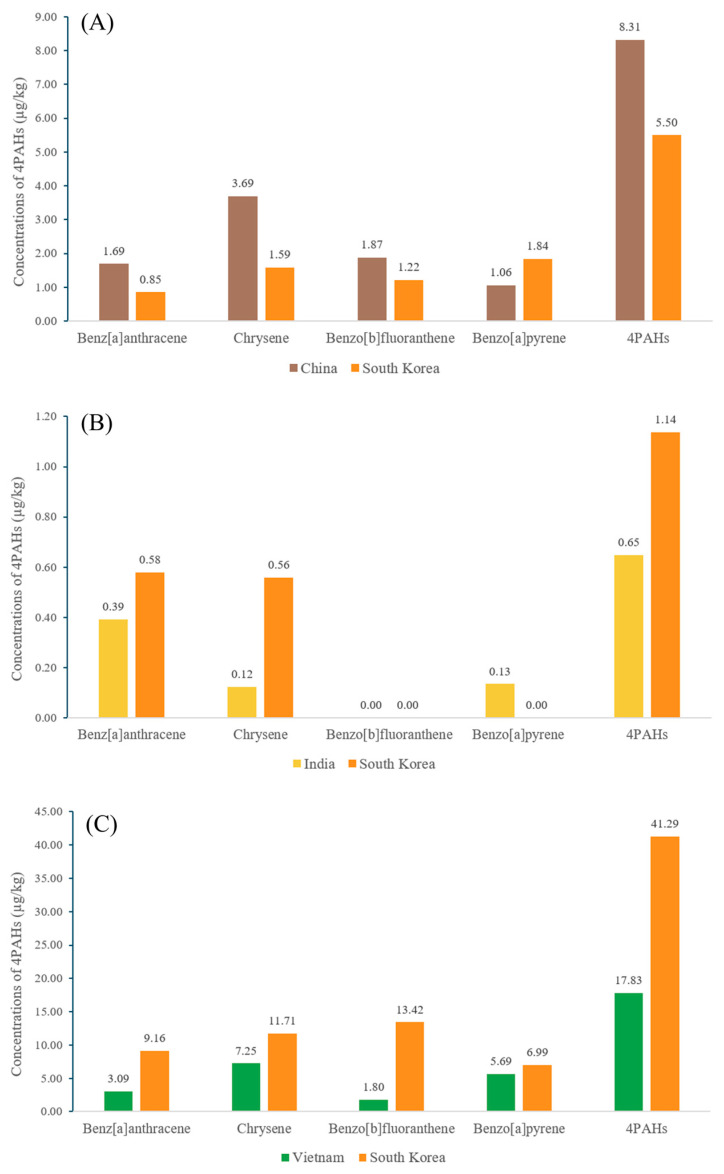
Comparison of average PAH concentrations in spice products by origin: (**A**) Sichuan pepper from South Korea and China, (**B**) Turmeric from South Korea and India, and (**C**) Cinnamon from South Korea and Vietnam.

To compare PAH concentrations in spice products distributed in South Korea by country of origin, three representative products were selected based on the inclusion of South Korean samples and a market distribution dominated by a few major countries, which allows for a clear comparative analysis (Figure 4). For Sichuan pepper, the average concentration of 4PAHs, excluding BaP, was consistently higher in samples from China than in those from South Korea. One possible contributor is the composition differences in fatty acids reported for the two varieties. Chinese Sichuan pepper has a high content of unsaturated fatty acids (82.2–85.3%), with linoleic acid comprising a significant portion (20.26%) [58,59]. In contrast, Sichuan peppers from Korea have a relatively higher proportion of oleic acid rather than linoleic acid [60]. During heating, linoleic acid forms aromatic rings via hydroperoxide and conjugated radicals, followed by dehydrogenation and ring expansion, leading to the formation of CHR and BaA [61]. At high temperatures, side-chain cleavage and radical recombination can also produce BbF [62]. Furthermore, a higher degree of unsaturation increases the formation of 4PAHs, resulting in greater PAH generation when linoleic acid is more abundant than oleic acid [63,64]. Therefore, the higher 4PAHs values in Chinese Sichuan pepper can be explained by this compositional difference, potentially interacting with processing steps. On the other hand, BaP is considered to be a late-stage product that is particularly sensitive to the intensity of heat treatment in addition to precursor composition. This is consistent with previous studies showing that low-molecular-weight PAHs can be converted into high-molecular-weight PAHs under harsher conditions through mechanisms like the Diels–Alder reaction [65,66,67].

In the case of turmeric, the PAH concentrations in Korean samples were observed to be higher than in Indian samples, except for BaP. According to the literature, Korean-grown turmeric is rich in hydrocarbon sesquiterpenes such as α-zingiberene and β-sesquiphellandrene, whereas Indian turmeric is dominated by ketonic sesquiterpenes like turmerones [68,69,70]. Sesquiterpenes have been reported to act as PAH precursors under harsh heating conditions [34]. Previous study indicates that the introduction of oxygenated functional groups tends to lower the formation of PAH precursors and the generation of PAHs [71,72]. It has also been reported within the terpenes that oxygenated terpenoids likely produce fewer PAHs than hydrocarbon terpenes [73]. Based on this evidence, it could be hypothesized that if high temperature exposure occurred during post-harvest handling, a matrix richer in hydrocarbon sesquiterpenes could enhance PAH formation upward relative to ketonic sesquiterpene-dominated matrices. However, external contamination and process design likely play a primary role in herb and spice product PAHs, with precursor composition acting as a conditional modifier under severe heat. As was the case with Sichuan pepper, BaP did not follow this tendency. This reaffirms that BaP formation is sensitive not only to precursor composition but also to the intensity of processing heat. In other words, even if Indian turmeric has a lower precursor potential, the possibility that it was exposed to more intense heat treatment conditions during post-harvest processes like drying cannot be excluded.

Cinnamon showed a different contamination pattern from the previous two samples. The single Korean cinnamon sample in this study had higher concentrations of all the PAHs than the average of the Vietnamese samples, although this cannot be generalized due to the sample limitation. Since cinnamon from both regions shares cinnamaldehyde as a major component, the influence of the cultivation environment rather than the raw material composition can be implicated [74,75,76]. Plants can accumulate PAHs from soil and the atmosphere, and soil PAH contamination is closely related to the level of industrial activity and urbanization [77,78,79]. For example, in China, higher soil PAH levels were observed in the eastern regions with high economic activity and population density compared to the western regions [80]. Indeed, significant differences in soil PAH contamination levels have been reported between industrial areas in South Korea and Vietnam. An average PAH concentration of 960 µg/kg has been reported in the soil of industrial cities in South Korea [79]. In contrast, a relatively lower residue of 692.6 µg/kg has been reported in the industrial areas of Vietnam [81]. Although the exact cultivation sites of the samples analyzed in this study are unknown, this difference in environmental background concentrations reported in the industrial areas of both countries could explain the results observed in this study.

**Figure 5:**
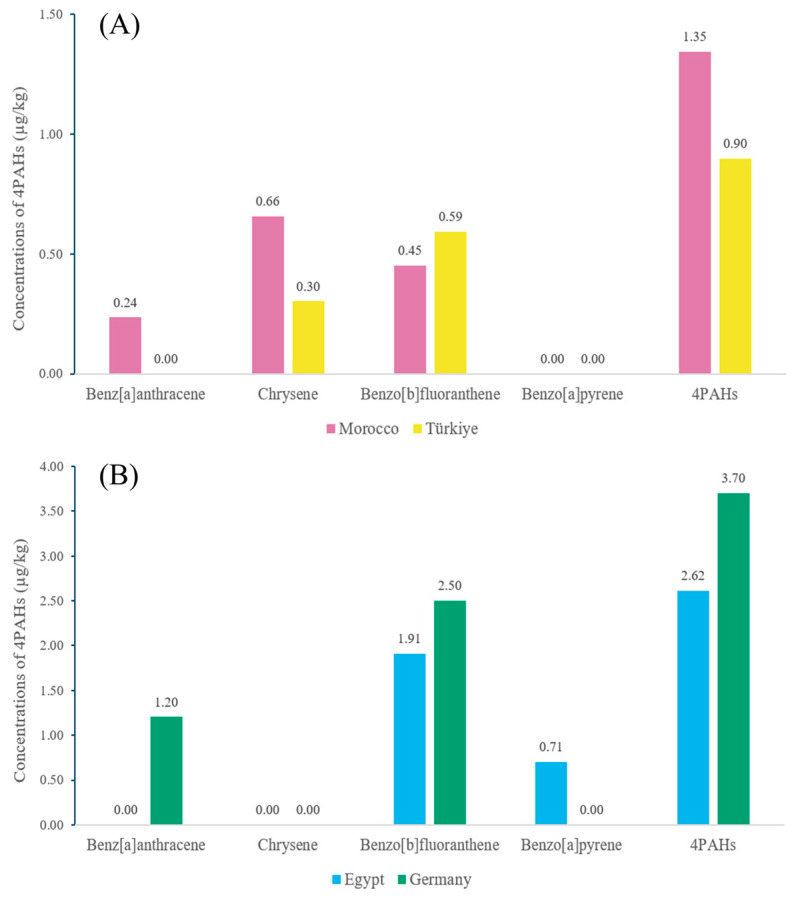
Comparison of average PAH concentrations in herb products by origin. (**A**) Rosemary from Morocco and Türkiye. (**B**) Parsley from Egypt and Germany.

For herbs distributed in South Korea, two representative commodities were selected for analysis based on having an origin composition concentrated in a few major exporting countries, which allows for a clear comparative analysis between nations (Figure 5). In the case of rosemary, samples from Morocco showed high levels of BaA and CHR, while samples from Türkiye showed high levels of BbF. Since rosemary from both countries has similar essential oil compositions, the difference in PAH concentrations can be explained by environmental background factors. As the molecular weight of PAHs increases, the dry deposition velocity decreases [82]. In dry climates, less rain means less wash-off of particle-bound PAHs, potentially increasing the persistence of BaA and CHR on plant surfaces. In this context, Morocco is drier than Türkiye with less precipitation, which could lead to less wash-off loss by rain, resulting in the relatively higher detection of BaA and CHR [83,84]. It is reported that the PAH of atmospheric environments in both countries have a high contribution from diesel emissions, which are closely related to traffic [85,86]. Traffic-dominated environments generally show a relatively high proportion of five-ring and six-ring PAHs in their emissions [87]. Therefore, a background exists in both countries for BbF to be detected as a significant contaminant. The particularly high level in the Turkish sample suggests the possibility of additional influences, such as specific local traffic volume, beyond common emission sources.

For parsley, PAH concentrations in German samples tended to be higher than in Egyptian samples. German parsley is reported to be dominated by monoterpenes such as myrcene and β-phellandrene, whereas Egyptian parsley is reported to have a high content of myristicin [88,89,90]. Monoterpenes, abundant in the German samples, can act as precursors that form the basic aromatic ring structure of PAHs at high temperatures in a manner similar to sesquiterpenes [91]. Furthermore, myristicin, which is abundant in the Egyptian samples, can act as an intermediate for PAH growth after its methylenedioxy ring is converted to a benzenediol like catechol during pyrolysis [92,93]. However, oxygenated intermediates such as catechol do not exclusively convert to PAHs. They can also undergo competitive pathways to form other oxygen-containing compounds like dibenzo-*p*-dioxin or dibenzofuran [94]. This is a factor that could affect the final PAH formation efficiency compared to precursors without oxygenated functional groups. In addition to chemical characteristics, environmental factors at the cultivation site may have caused the difference in contamination levels. Atmospheric PAH concentrations are known to increase significantly in winter due to the surge in heating fuel consumption [95]. Germany, with its relatively colder and longer winters, may have exposed plants to higher levels of accumulated atmospheric PAHs during their growth or drying periods compared to the mild climate of Egypt.

It is important to note that the descriptions of chemical compositions and country-of-origin environmental contexts of herbs and spices presented in this section were inferred from prior literature, not empirically verified in this study. Accordingly, any association between these factors and PAH levels should be interpreted with caution.

### 3.4. Risk Assessment of PAHs in Herbs and Spices

The carcinogenicity and mutagenicity of PAHs were assessed using TEQ (Table 6). TEQ is directly associated with carcinogenic potential. For the calculation of TEQ_Σ4PAHs_, TEFs of 0.1, 0.01, 0.1, and 1 were applied to BaA, CHR, BbF, and BaP. Across all samples, TEQ_BaP_ and TEQ_Σ4PAHs_ were 1.81 µg/kg and 0.52 µg/kg, respectively. Among individual samples, oregano showed the highest TEQ_Σ4PAHs_ levels among the herbs, with a value of 7.50 µg/kg, followed by the spice cinnamon, which showed 6.87 µg/kg. The TEQ_Σ4PAHs_ is expected to be primarily driven by BaP, with BbF and BaA acting as secondary contributors and CHR making only a minor contribution. Accordingly, even modest BaP levels can disproportionately elevate the TEQ_Σ4PAHs_, which explains the relatively high TEQ_Σ4PAHs_ values observed for oregano and cinnamon in the present study [96]. Turmeric displayed the lowest levels, while nutmeg and mustard also showed relatively low values.

By category, herbs generally showed higher values than spices. The mean values for herbs were 2.18 µg/kg for TEQ_BaP_ and 2.46 µg/kg for TEQ_Σ4PAHs_, whereas the corresponding means for spices were 1.50 and 1.75 µg/kg, respectively. The difference between herbs and spices was largely driven by the high levels of these values observed in oregano. However, other herbs, such as rosemary and bay leaf, also tended to show higher values than the spice group. From a risk-management perspective, products with a high TEQ should be prioritized for targeted mitigation, such as controlling drying/smoking parameters and implementing periodic verification testing. In contrast, low-TEQ products can remain under routine surveillance.

### 3.5. Dietary Risk Characterization of 4PAHs in Herbs and Spices

MOE is a useful indicator for setting risk reduction priorities by comparing human intake levels of hazardous substances with appropriate reference points. In this study, TEQs for BaP and Σ4PAHs and MOEs based on the mean and 95th-percentile intakes were calculated for 11 herbs and spices (Table 6). Among the samples, black pepper exhibited the lowest MOE values, with 4.67 × 10^7^ for BaP and 1.74 × 10^8^ for Σ4PAHs, indicating a relatively high level of concern. Turmeric followed, with MOE values of 1.42 × 10^8^ for BaP and 4.82 × 10^8^ for Σ4PAHs. In contrast, nutmeg showed the highest MOE values, with 1.96 × 10^11^ for BaP and 4.90 × 10^11^ for Σ4PAHs. Within the same samples, MOE values based on Σ4PAHs were generally higher than those based on BaP. This suggests that under the BMDL_10_ weighting scheme applied in this study, the BaP index provided a more conservative safety margin than the Σ4PAHs index. The MOEs based on the 95th percentile intake decreased compared to the mean intake, as anticipated. For the BaP-based assessment, cinnamon (1.39 × 10^6^) and turmeric (4.15 × 10^6^) showed the lowest values, whereas most other samples exceeded. Similarly, the MOEs for ∑4PAHs remained high overall, except for cinnamon (6.07 × 10^6^), which was below. All MOE values, including high-intake across samples, exceeded 10^6^, which is far above the reference threshold of 10^4^. In general, an MOE above 10^4^ is considered to indicate a low public health concern. The low dietary contribution of herbs and spices to overall PAH exposure is primarily due to their very small daily consumption. Assuming an adult body weight of 66.83 kg and applying the TEQ_BaP_ = 6.18 µg/kg and TEQ_Σ4PAHs_ = 6.87 µg/kg for cinnamon, the MOE can reach lower than 10^4^ when more than 68 g/day of cinnamon is consumed for BaP, and about 297 g/day for Σ4PAHs. These intake levels are tens to hundreds of times higher than the typical daily consumption of herbs and spices, indicating that the actual risk under realistic dietary conditions is negligible.

The differences between the products are attributable to a combination of TEQ values and dietary consumption patterns. For instance, black pepper exhibited the lowest mean MOE. This was attributed to its widespread use and high frequency of consumption, rather than an exceptionally high TEQ. On the other hand, nutmeg displayed a very large MOE due to both a low TEQ and low consumption frequency. Cinnamon showed the lowest MOE in the high-consumption scenario, a result interpreted as the combined effect of its high intake, particularly through desserts and beverages, and its TEQ. The results of this study are also consistent with previous reports on Belgian retail herbs and spices, in which MOE values consistently exceeded 20,000 under the assumption of average contamination levels [97]. Therefore, the public health concern associated with 4PAHs exposure from herbs and spices is considered low.

## 4. Conclusions

This study quantified 4PAHs in 110 herb and spice products marketed in South Korea using a validated GC-MS method. The analysis revealed that cinnamon and oregano exhibited the highest BaP concentrations among the tested spices and herbs. Across all products, mean PAH concentrations were generally below EU maximum limits. However, the upper quartile of BaP in oregano reached 10.84 µg/kg, exceeding the EU BaP maximum level (10 µg/kg) and suggesting a non-compliance risk. Nevertheless, MOE-based risk characterization remained above 10,000 across evaluated intake scenarios, indicating low public-health concern at current consumption. Given that several major markets, including Australia, China, and the United States, lack product-specific PAH limits for herbs and spices, our results provide baseline evidence to inform risk-based monitoring and development of standards.

Comparison with international studies reveals that while contamination for certain herbs and spices is recurrent across various regions, the PAHs for the same kind of product can differ significantly by country. This is interpreted as the result of a complex effect of factors, including the composition of essential oils, the climate and precipitation of the origin, geographical and industrial activity density, and the fuels and process conditions used for drying. As a study conducted with final products available on the market, this research could not quantitatively distinguish the contribution of each supply chain stage to the observed PAH contamination. Furthermore, while the analytical method demonstrated good recovery rates, the possibility of residual matrix effects due to the diverse characteristics of the samples cannot be completely excluded. Future studies are necessary to quantify PAHs using samples with clearly characterized cultivation environments and processing conditions, coupling controlled processing with pre–post-analyses to resolve stage-specific contributions to PAH formation.

These findings suggest the need for targeted observation and the phased introduction of scientific management standards for herbs and spices distributed in South Korea. Commodities with the potential to exceed international limits should be determined as high-risk groups and managed intensively with increased inspection frequency. Given the origin-specific variations for the same product, it is necessary to strengthen inspections at the import stage for items from regions with environmental concerns. Establishing review levels with reference to international standards and gradually expanding the scope of legal limits as more data is accumulated would enhance regulatory efficiency and international comparability.

## Figures and Tables

**Table 1:** Evaluation of LOD, LOQ, and linearity of PAHs analysis in herb and spice products.

Sample Type	Compounds	Target Ion	Qualitative Ions	Retention Time	Equation (y = ax + b)	Linearity (R^2^)	LOD ^(1)^ (μg/kg)	LOQ ^(2)^ (μg/kg)
Spice (mustard)	Benz[a]anthracene	228	226, 229	22.793	y = 0.0085x − 0.0013	1.000	0.08	0.24
	Chrysene			23.246	y = 0.0115x + 0.0010	0.998	0.15	0.46
	Benzo[b]fluoranthene	252	250, 253	30.462	y = 0.0158x + 0.0037	0.999	0.18	0.55
	Benzo[a]pyrene			32.161	y = 0.0109x + 0.0016	0.999	0.12	0.37
Herb (parsley)	Benz[a]anthracene	228	226, 229	22.793	y = 0.0082x − 0.0014	0.999	0.17	0.51
	Chrysene			23.246	y = 0.0113x + 0.0010	1.000	0.11	0.32
	Benzo[b]fluoranthene	252	250, 253	30.462	y = 0.0150x + 0.0021	1.000	0.13	0.41
	Benzo[a]pyrene			32.161	y = 0.0097x + 0.0018	0.999	0.15	0.46

^(1)^ LOD (μg/kg) based on signal-to-noise ratio as S/N = 3.3; ^(2)^ LOQ (μg/kg) based on signal-to-noise ratio as S/N = 10.

**Table 2:** Evaluation of recovery for PAHs in herb and spice products.

Sample Type	Compounds	Recovery (%)				
		1 μg/kg	2 μg/kg	5 μg/kg	10 μg/kg	20 μg/kg
Spice (mustard)	Benz[a]anthracene	88.6 ± 1.2	98.4 ± 1.1	101.0 ± 1.2	116.2 ± 0.9	116.5 ± 0.2
	Chrysene	102.1 ± 1.3	98.6 ± 0.4	107.6 ± 1.3	103.3 ± 1.0	98.1 ± 1.3
	Benzo[b]fluoranthene	96.3 ± 0.5	99.9 ± 0.6	93.0 ± 1.2	97.7 ± 0.7	93.5 ± 1.4
	Benzo[a]pyrene	95.6 ± 1.0	96.1 ± 0.6	100.0 ± 1.5	112.2 ± 0.4	109.0 ± 1.2
Herb (parsley)	Benz[a]anthracene	109.3 ± 0.7	97.2 ± 0.8	94.2 ± 0.3	104.1 ± 1.4	112.9 ± 1.3
	Chrysene	108.3 ± 1.4	101.1 ± 1.1	97.7 ± 1.1	97.6 ± 0.9	96.5 ± 1.1
	Benzo[b]fluoranthene	94.5 ± 0.3	90.7 ± 0.5	85.0 ± 0.0	89.4 ± 0.6	89.0 ± 0.9
	Benzo[a]pyrene	91.4 ± 1.2	97.7 ± 0.9	95.0 ± 0.3	96.4 ± 0.8	99.8 ± 1.2

**Table 3:** Evaluation of method accuracy and precision for PAHs in herb and spice products over intra-day and inter-day runs.

Sample Type	Compounds		Intra-Day (*n* = 3)		Inter-Day (*n* = 3)	
		μg/kg	Accuracy (%) ^(1)^	RSD (%) ^(2)^	Accuracy (%)	RSD (%)
Spice	Benz[a]anthracene	1	88.6	1.7	88.1	1.3
		2	98.6	1.5	98.2	0.5
		5	101.2	1.5	100.7	0.5
		10	116.2	0.9	115.8	0.8
		20	116.6	0.3	116.6	0.1
	Chrysene	1	102.1	1.6	102.6	1.2
		2	98.6	0.5	98.5	0.5
		5	107.4	1.7	107.4	0.9
		10	103.4	1.3	103.7	1.1
		20	98.1	1.7	97.5	1.5
	Benzo[b]fluoranthene	1	96.3	0.7	96.1	0.5
		2	99.9	0.8	99.7	0.4
		5	92.9	1.7	93.4	1.0
		10	97.8	1.0	97.9	0.6
		20	93.4	2.0	93.6	0.6
	Benzo[a]pyrene	1	95.5	1.4	95.1	1.2
		2	96.1	0.8	96.3	0.4
		5	99.8	2.0	99.5	1.5
		10	112.3	0.5	112.4	0.4
		20	109.0	1.4	108.5	1.1
Herb	Benz[a]anthracene	1	109.4	0.8	109.6	0.6
		2	97.2	1.1	96.9	0.9
		5	94.2	0.5	94.3	0.1
		10	104.4	1.7	104.3	1.2
		20	112.6	1.5	112.6	1.1
	Chrysene	1	108.1	1.8	107.8	1.0
		2	101.1	1.3	100.7	1.0
		5	97.9	1.4	97.5	0.3
		10	97.8	1.2	97.6	0.6
		20	96.8	1.5	96.7	0.9
	Benzo[b]fluoranthene	1	94.5	0.5	94.4	0.3
		2	90.7	0.8	90.7	0.3
		5	85.0	0.0	85.0	0.0
		10	89.3	0.9	89.2	0.7
		20	89.1	1.3	88.7	0.5
	Benzo[a]pyrene	1	91.3	1.7	91.8	1.1
		2	97.7	1.2	98.1	1.0
		5	95.0	0.4	95.2	0.3
		10	96.5	1.1	96.1	0.5
		20	99.6	1.7	100.2	0.4

^(1)^ Accuracy (%) = [1 − (mean concentration measured − concentration spiked)/concentration spiked] ×100. ^(2)^ RSD (relative standard deviation, %) = (standard deviation/mean) × 100.

**Table 4:** Quantification of PAHs detected in herb and spice products by GC-MS.

Sample Type	Sample	*N* ^(1)^	Mean ± SD (μg/kg)				
			Benz[a]anthracene	Chrysene	Benzo[b]fluoranthene	Benzo[a]pyrene	Σ4PAHs
Spice	Mustard	10	0.18 ± 0.45	0.42 ± 1.12	0.15 ± 0.49	0.29 ± 0.92	0.26 ± 0.77
	Nutmeg	10	1.05 ± 0.81	1.09 ± 0.95	0.00 ±0.00	0.12 ± 0.28	0.56 ± 0.80
	Black pepper	10	1.86 ± 1.98	3.13 ± 2.70	0.65 ± 1.86	0.93 ± 1.56	1.64 ± 2.22
	Sichuan pepper	10	1.36 ± 1.87	2.85 ± 4.66	1.61 ± 2.11	1.37 ± 2.44	1.80 ± 2.93
	Cinnamon	10	3.39 ± 4.86	6.98 ± 5.27	2.78 ± 4.30	6.18 ± 3.46	4.83 ± 4.71
	Turmeric	10	0.39 ± 0.44	0.20 ± 0.42	0.00 ±0.00	0.09 ± 0.20	0.17 ± 0.34
Herb	Basil	10	0.83 ± 0.58	0.93 ± 0.83	1.30 ± 2.46	1.75 ± 2.84	1.23 ± 1.90
	Oregano	10	0.32 ± 0.50	1.10 ± 1.31	3.18 ± 3.26	7.13 ± 6.68	2.94 ± 4.51
	Parsley	10	0.35 ± 0.50	1.76 ± 1.77	0.00 ±0.00	0.48 ± 1.52	0.65 ± 1.33
	Rosemary	10	0.07 ± 0.22	0.36 ± 0.69	0.61 ± 1.03	0.77 ± 2.43	0.45 ± 1.34
	Bay leaves	10	5.12 ± 5.33	7.61 ± 6.50	0.88 ± 1.07	0.79 ± 1.05	3.60 ± 5.04

^(1)^ *N*: number of herb and spice samples used for the experiment.

**Table 5:** Detection frequency and median (Q1–Q3) concentrations of PAHs in herb and spice products.

Sample Type	Sample	Benz[a]anthracene		Chrysene		Benzo[b]fluoranthene		Benzo[a]pyrene		Σ4PAHs	
		Detection Rate (%)	Median (Q1–Q3) (μg/kg)	Detection Rate (%)	Median (Q1–Q3) (μg/kg)	Detection Rate (%)	Median (Q1–Q3) (μg/kg)	Detection Rate (%)	Median (Q1–Q3) (μg/kg)	Detection Rate (%)	Median (Q1–Q3) (μg/kg)
Spice	Mustard	20.0	0.00 (0.00–0.00)	20.0	0.00 (0.00–0.00)	10.0	0.00 (0.00–0.00)	10.0	0.00 (0.00–0.00)	15.0	0.00 (0.00–0.00)
	Nutmeg	80.0	1.01 (0.41–1.62)	70.0	1.08 (0.16–1.66)	0.0	0.00 (0.00–0.00)	20.0	0.00 (0.00–0.00)	42.5	0.00 (0.00–0.98)
	Black pepper	80.0	1.66 (0.85–1.82)	90.0	2.93 (1.88–3.27)	20.0	0.00 (0.00–0.00)	60.0	0.47 (0.00–0.56)	62.5	0.63 (0.00–2.51)
	Sichuan pepper	60.0	0.71 (0.00–1.31)	60.0	1.03 (0.00–2.69)	40.0	0.00 (0.00–3.63)	40.0	0.00 (0.00–1.48)	50.0	0.31 (0.00–3.03)
	Cinnamon	80.0	1.62 (1.20–1.95)	90.0	5.88 (4.16–7.59)	70.0	0.88 (0.14–3.94)	90.0	7.02 (5.26–8.74)	82.5	4.29 (0.98–7.15)
	Turmeric	50.0	0.25 (0.00–0.75)	20.0	0.00 (0.00–0.00)	0.0	0.00 (0.00–0.00)	20.0	0.00 (0.00–0.00)	22.5	0.00 (0.00–0.00)
Herb	Basil	80.0	0.75 (0.63–1.24)	70.0	1.22 (0.16–1.47)	40.0	0.00 (0.00–1.02)	40.0	0.00 (0.00–2.26)	57.5	0.69 (0.00–1.42)
	Oregano	40.0	0.00 (0.00–0.66)	50.0	0.48 (0.00–1.96)	70.0	2.25 (0.39–4.76)	70.0	6.95 (0.64–10.84)	23.0	0.91 (0.00–3.50)
	Parsley	40.0	0.00 (0.00–0.67)	70.0	1.14 (0.22–2.80)	0.0	0.00 (0.00–0.00)	10.0	0.00 (0.00–0.00)	12.0	0.00 (0.00–0.76)
	Rosemary	10.0	0.00 (0.00–0.00)	30.0	0.00 (0.00–0.33)	30.0	0.00 (0.00–1.02)	10.0	0.00 (0.00–0.00)	20.0	0.00 (0.00–0.00)
	Bay leaves	90.0	3.00 (2.10–6.06)	100.0	5.78 (4.73–8.88)	60.0	0.60 (0.00–1.34)	40.0	0.00 (0.00–1.79)	29.0	2.03 (0.00–4.73)

**Table 6:** Toxic equivalent quantity (TEQ) and margin of exposure (MOE) of PAHs in herb and spice products.

Sample Type	Sample	TEQ_BaP_ (μg/kg)	TEQ_Σ4PAHs_ (μg/kg)	MOE—Average Dietary Exposure		MOE—95th Percentile Dietary Exposure	
		BaP	Σ4PAHs	BaP	Σ4PAHs	BaP	Σ4PAHs
Spice	Mustard	0.29	0.33	4.97 × 10^9^	2.14 × 10^10^	1.17 × 10^8^	5.04 × 10^8^
	Nutmeg	0.12	0.24	1.96 × 10^11^	4.90 × 10^11^	6.49 × 10^7^	1.62 × 10^8^
	Black pepper	0.93	1.22	4.67 × 10^7^	1.74 × 10^8^	4.57 × 10^7^	1.70 × 10^8^
	Sichuan pepper	1.37	1.70	1.23 × 10^9^	4.82 × 10^9^	8.57 × 10^7^	3.37 × 10^8^
	Cinnamon	6.18	6.87	1.00 × 10^9^	4.38 × 10^9^	1.39 × 10^6^	6.07 × 10^6^
	Turmeric	0.09	0.14	1.42 × 10^8^	4.82 × 10^8^	4.15 × 10^6^	1.41 × 10^7^
Herb	Basil	1.75	1.98	1.62 × 10^9^	6.98 × 10^9^	2.02 × 10^7^	8.70 × 10^7^
	Oregano	7.13	7.50	7.67 × 10^9^	3.55 × 10^10^	6.56 × 10^8^	3.03 × 10^9^
	Parsley	0.48	0.54	3.06 × 10^9^	1.34 × 10^10^	7.89 × 10^7^	3.45 × 10^8^
	Rosemary	0.77	0.84	3.74 × 10^9^	1.66 × 10^10^	2.33 × 10^8^	1.03 × 10^9^
	Bay leaves	0.79	1.46	2.66 × 10^9^	6.94 × 10^9^	2.11 × 10^7^	5.51 × 10^7^

## Data Availability

The original contributions presented in this study are included in the article. Further inquiries can be directed to the corresponding author.
